# Association of Ischemic and Bleeding Events With Mortality Among Patients in Sweden With Recent Acute Myocardial Infarction Receiving Antithrombotic Therapy

**DOI:** 10.1001/jamanetworkopen.2022.20030

**Published:** 2022-08-29

**Authors:** Moa Simonsson, Joakim Alfredsson, Karolina Szummer, Tomas Jernberg, Peter Ueda

**Affiliations:** 1 Department of Clinical Sciences, Cardiology, Karolinska Institutet, Danderyd Hospital, Stockholm, Sweden; 2 Department of Health, Medicine and Caring Sciences and Department of Cardiology, Linköping University, Linköping, Sweden; 3 Department of Medicine, Karolinska Institutet, Cardiology, Karolinska University Hospital, Stockholm, Sweden; 4 Clinical Epidemiology Division, Department of Medicine, Solna, Karolinska Institutet, Stockholm, Sweden

## Abstract

**Importance:**

Antithrombotic treatment after myocardial infarction (MI) should be individualized based on the patient’s risk of ischemic and bleeding events. Uncertainty remains regarding the relative prognostic importance of the 2 types of events, and further study would be useful.

**Objectives:**

To compare the association of ischemic vs bleeding events with mortality in patients with a recent MI and assess whether the relative mortality risk for the 2 types of events has changed over the past 2 decades.

**Design, Setting, and Participants:**

A cohort study based on nationwide registers in Sweden, 2012-2017, was conducted. Data were analyzed between July 2021 and May 2022. Patients with MI who were discharged alive with antithrombotic therapy (antiplatelet therapy or oral anticoagulation) were included in the analysis.

**Main Outcomes and Measures:**

The incidence of a first ischemic event (hospitalization for MI or ischemic stroke) or bleeding event (hospitalization with bleeding) up to 1 year after discharge and the mortality risk up to 1 year after each type of event were assessed. Cox proportional hazards regression models were used to estimate adjusted hazard ratios (aHRs) for 1-year mortality after an ischemic and bleeding event vs no event, and after an ischemic vs bleeding event. Adjusted HRs for mortality after ischemic vs bleeding events were compared among patients discharged in 1997-2000, 2001-2011, and 2012-2017.

**Results:**

Of 86 736 patients discharged after MI in 2012-2017 (median age, 71 [IQR, 62-80] years; 57 287 [66.0%] men), 4039 individuals experienced a first ischemic event (5.7 per 100 person-years) and 3399 experienced a first bleeding event (4.8 per 100 person-years). The mortality rate was 46.2 per 100 person-years after an ischemic event and 27.1 per 100 person-years after a bleeding event. The aHR for 1-year mortality vs no event was 4.16 (95% CI, 3.91-4.43) after an ischemic event and 3.43 (95% CI, 3.17-3.71) after a bleeding event. When the 2 types of events were compared, the aHR was 1.27 (95% CI, 1.15-1.40) for an ischemic vs bleeding event. There was no statistically significant difference in the aHR of an ischemic vs bleeding event in 1997-2000, 2001-2011, and 2012-2017.

**Conclusions and Relevance:**

In this nationwide cohort study of patients with a recent MI, postdischarge ischemic events were more common and associated with higher mortality risk compared with bleeding events.

## Key Points

**Question:**

What is the association of an ischemic event vs a bleeding event with mortality among patients with myocardial infarction (MI)?

**Findings:**

In this cohort study of 86 736 patients in Sweden with a recent MI. Recurrent ischemic events were more common and associated with higher risk of 1-year mortality compared with bleeding events.

**Meaning:**

The findings of this study suggest that, although strategies to individualize intensity and duration of antithrombotic treatment after MI typically assign similar weight to bleeding and ischemic events, the associated mortality risk may be higher for ischemic events.

## Teaser

This cohort study compares mortality associated with recurrent ischemic vs bleeding events during antithrombotic therapy among patients in Sweden with myocardial infarction.

## Introduction

Antithrombotic treatment is used to reduce the risk of recurrent ischemic events in patients with acute myocardial infarction (MI). Although most patients receive dual antiplatelet therapy (DAPT) with aspirin and a P2Y12 inhibitor,^1^ treatment options for those with concomitant atrial fibrillation/flutter or other indications for oral anticoagulation (OAC) include triple therapy with DAPT and OAC or dual antithrombotic treatment with a single antiplatelet agent and OAC.^2^ The ischemic risk reduction conferred by these treatments comes at the expense of an increased risk of bleeding. European^2^ and US^3^ guidelines therefore recommend individualized antithrombotic therapy based on the patient’s ischemic and bleeding risk.^4,5,6^ For example, the duration of DAPT can be prolonged^7,8^ for patients with high ischemic risk and without high bleeding risk, and DAPT can be shortened or deescalated^9,10^ for those with a high bleeding risk and lower ischemic risk.^11,12,13^

Although the strategies to individualize antithrombotic treatment are based on calculations that give equal weight to ischemic and bleeding events,^14,15,16^ uncertainty remains regarding the relative importance of ischemic vs bleeding events among patients with a recent MI. Several studies have assessed the mortality associated with recurrent MI and bleeding in patients with acute coronary syndrome or undergoing percutaneous coronary intervention (PCI).^17^ However, most of these studies were based on randomized clinical trials^13,18,19,20,21^ with uncertain generalizability to patients seen in routine clinical practice, assessed events occurring beyond 1 year after PCI when DAPT has been terminated for most patients,^13^ or compared the mortality risk associated with recurrent MI vs bleeding, although ischemic stroke is an important ischemic event whose risk can be modified using antithrombotic treatment.^13^ In addition, during the past decades, reperfusion therapy, improved secondary prevention,^22,23^ and high-sensitivity cardiac troponins have been introduced; these advances may limit the applicability of previous studies to contemporary clinical practice. Data on the occurrence of and the mortality associated with ischemic and bleeding events may inform the bleeding vs ischemic risk trade-off for treatments of patients with MI.

In this study, we used nationwide registers in Sweden to compare the association of ischemic vs bleeding events with 1-year mortality in patients with a recent MI who received antithrombotic therapy. We also examined how the association of an ischemic vs bleeding event with mortality had changed over the past 2 decades.

## Methods

### Data Sources

In this cohort study, we used data from the Swedish Web-System for Enhancement and Development of Evidence-Based Care in Heart Disease Evaluated According to Recommended Therapies (SWEDEHEART),^24^ a national register including patients with acute MI admitted to a coronary care unit or other specialized inpatient facilities in Sweden. We linked these data to the National Patient Register (NPR)^25^ (data on comorbidities and study outcomes) and the Swedish Population Registers^26^ (vital status and date of death); details regarding the data sources are provided in the eMethods and eTable 1 in the Supplement. The study was approved by the ethics committee in Stockholm, Sweden. No written informed consent was required because data were deidentified. This study followed the Strengthening the Reporting of Observational Studies in Epidemiology (STROBE) reporting guideline.

### Study Population

**Figure 1.**
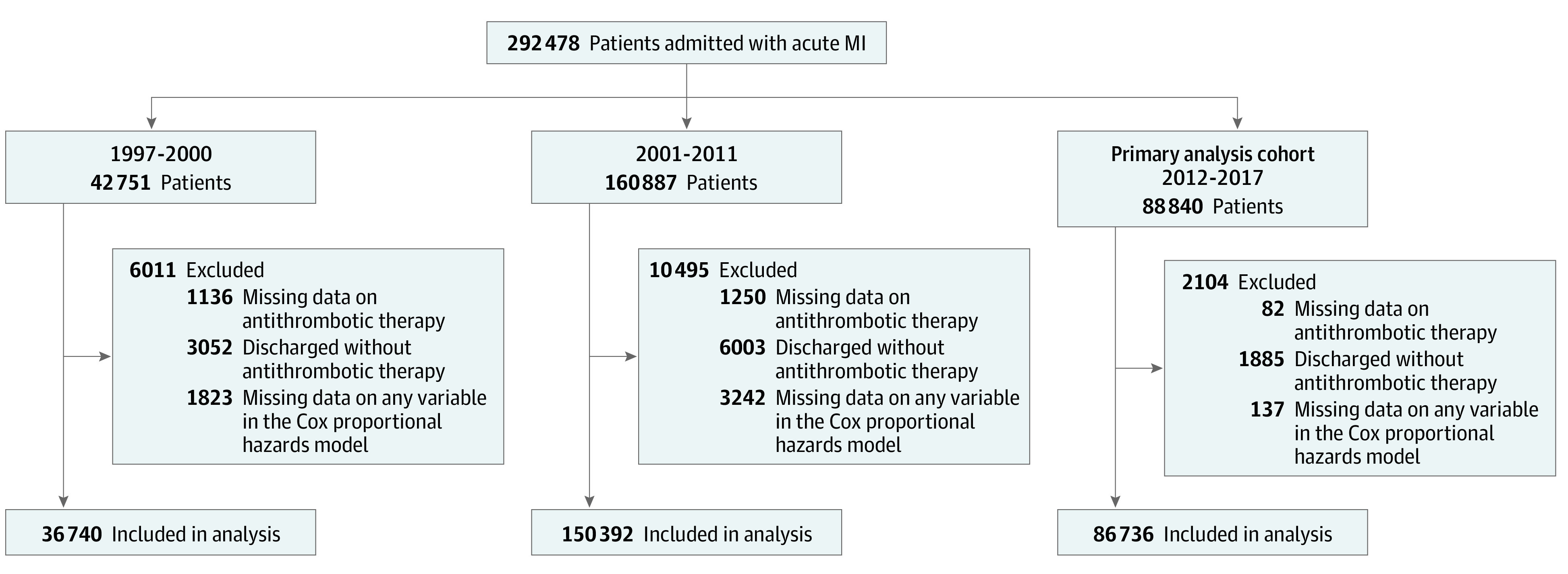
Patients Discharged After Myocardial Infarction (MI) Between January 1997 and December 2017 A total of 49 408 patients had more than 1 hospital admission for MI during the study period. For these patients, 1 admission was randomly selected. The primary analysis was performed in the cohort discharged from January 1, 2012, to December 31, 2017.

We included all patients aged 18 years or older with acute MI (*International Statistical Classification of Diseases, 10th Revision* [*ICD-10*] code I21) who were enrolled in the SWEDEHEART registry from January 1, 1997, to December 31, 2017, and were discharged alive. For patients with multiple admissions during the study period, 1 admission was randomly selected. We excluded patients discharged without any antithrombotic treatment (aspirin, P2Y12 inhibitor, or oral anticoagulant) or with missing data on any of the covariates used in the primary analysis (eTable 1 in the Supplement); in 2012-2017, 10 of 26 covariates had missing data, and the proportion missing per variable was less than 0.1% (Figure 1; eTable 1 in the Supplement).

### Definition of Ischemic and Bleeding Events

We considered ischemic events, defined as MI and ischemic stroke, and bleeding events occurring within 365 days from discharge because the risk of events occurring during the first year after discharge is typically used to guide treatment decisions.^1,2,3^ Myocardial infarction was defined as hospitalization for MI registered in the SWEDEHEART registry (days 2-30 after discharge) or in the NPR (days 31-365 after discharge) with *ICD-10* code I21 as primary diagnosis. Events registered in SWEDEHEART during the first 2 days after discharge and in the NPR during the first 30 days after discharge were not considered to avoid including multiple entries of the index MI event. Ischemic stroke (days 1-365 after discharge) was defined as hospitalization registered in the NPR with *ICD-10* code I63 as the primary or secondary diagnosis (eTable 2 in the Supplement). Bleeding was defined as hospitalization registered in the NPR with an *ICD-10* code for bleeding (eTable 3 in the Supplement) days 1 to 365 after discharge as primary or secondary diagnosis.^27^

For patients experiencing both an ischemic and bleeding event, only the first event was considered. If both types of events occurred on the same day, only the ischemic event was considered, because few patients experienced ischemic and bleeding events on the same day (159 of 4039 patients experiencing a first ischemic event in the primary analysis) and as we considered it more likely that a bleeding event occurred immediately after an ischemic event (potentially due to the treatment) than an ischemic event occurring immediately after a bleeding event.

### Data Analysis

Patients were categorized based on their date of discharge into 3 time-periods that were selected to broadly reflect different paradigms for treatment of patients with MI: 1997-2000 (before routine PCI and DAPT were introduced), 2001-2011 (when routine PCI and DAPT were implemented) and 2012-2017 (contemporary practice with wide use of more potent P2Y12 inhibitors as part of DAPT).^22,23^ In the primary analysis, only data from 2012-2017 were used. In the secondary analysis assessing potential changes in the association of mortality with ischemic vs bleeding events, data from all 3 time periods were used.

We described the cumulative incidence of ischemic and bleeding events in the first 365 days after discharge from the index MI using Kaplan-Meier methods. Patients were followed up from discharge until an ischemic or bleeding event, death, end of the study (December 31, 2017), or 365 days after discharge.

#### Primary Analyses

Two approaches were used to assess the association of ischemic and bleeding events with mortality. First, we used a time-varying exposure definition in which each person-day of follow-up was categorized into 1 of 3 mutually exclusive categories (after an ischemic event, after a bleeding event, and no event); this method allows for patients to change exposure category during follow-up to estimate risk of the outcome associated with each exposure category.^28^ We used Cox proportional hazards regression models with all-cause death as the outcome to estimate crude hazards ratios (HRs) and adjusted HRs (aHRs) for 1-year mortality associated with an ischemic and bleeding event compared with no event. In this analysis patients were followed up from discharge after their index MI to death, 730 days after discharge, 365 days after an ischemic or bleeding event, or end of the study (December 31, 2017). Patients who experienced their first bleeding or ischemic event after 365 days from discharge were censored at the time of this event. The adjusted Cox proportional hazards regression model was adjusted for sex, age, year of discharge, comorbidities (as measured at the index MI), and discharge medications, including antithrombotic treatment (eTable 1 in the Supplement).

Next, we restricted the analyses to patients experiencing an ischemic or bleeding event within 365 days after discharge from the index MI. Starting from the day of the ischemic or bleeding event, patients were followed up until death, end of the study (December 31, 2017), or 365 days after the event. We used Cox proportional hazards regression models to estimate crude HRs and aHRs for 1-year mortality associated with an ischemic event compared with a bleeding event. In addition to the variables listed in eTable 1 in the Supplement, the adjusted model included a covariate for time from discharge to the ischemic or bleeding event.

#### Secondary Analyses

To assess whether the risk of mortality after an ischemic vs bleeding event had changed across time periods, data from all 3 periods (1997-2000, 2001-2011, and 2012-2017) were used. In each time period, we calculated the crude HRs and aHRs of mortality for an ischemic vs bleeding event among patients who experienced either type of event. We tested for differences in the HR by using an interaction term between time period and ischemic vs bleeding event.

#### Sensitivity Analyses

One sensitivity analysis was performed to assess the potential association between additional variables, including smoking status, hemoglobin level, and estimated glomerular filtration rate, and the results. In this analysis, we included only patients with data available on the additional variables and performed the primary analyses with and without adjustment for the additional variables. In a post hoc sensitivity analysis, we categorized patients experiencing an ischemic event and a bleeding event on the same day they were exposed to a bleeding event.

#### Post Hoc Analyses

Three post hoc analyses were performed: (1) the ischemic event definition was restricted to include only recurrent MI, (2) separate analyses were performed for those discharged with OAC and without OAC, and (3) potential differences in short-term mortality were assessed for the 2 types of events, HRs were estimated for mortality within 30 days after an ischemic vs bleeding event.

### Statistical Analysis

Hazard ratios are presented with 95% CIs. A significance level *P* < .05 was used and all tests were 2-sided. All statistical analyses were performed using Stata, version 15 (StataCorp LLC).

## Results

### Study Population

Characteristics at the index MI of patients discharged in 2012-2017 (n = 86 736) are reported in Table 1. Median age was 71 (IQR, 62-80) years, 29 449 (34.0%) were women, 57 287 (66.0%) were men, and 29 291 (33.8%) presented with ST-elevation MI. Most patients received DAPT at discharge (69 217 [79.8%]), with ticagrelor being the most common P2Y12 inhibitor (48 760 [56.2%]); 11 461 patients (13.2%) received single antiplatelet therapy, 9935 (11.5%) received an OAC, and 8157 (9.4%) received either single or dual antiplatelet therapy combined with an OAC.

**Table 1.** Characteristics at Index MI of Patients Who Were Discharged Alive After an MI in Sweden in 2012-2017

Characteristic	Patients, No. (%)			
	All (N = 86 736)	By event status during 365 d after discharge		
		Ischemic event (n = 4039)	Bleeding event (n = 3399)	No event (n = 79 298)
Demographic				
Age, median (IQR), y^a^	71 (62-80)	79 (70-86)	76 (68-83)	70 (62-79)
Sex				
Female	29 449 (34.0)	1666 (42.2)	1093 (32.2)	26 690 (33.7)
Male	57 287 (66.0)	2373 (58.8)	2306 (67.8)	52 608 (66.3)
STEMI	29 291 (33.8)	879 (21.8)	1061 (31.2)	27 351 (34.5)
Medical history				
Hypertension	51 546 (59.4)	3150 (78.0)	2290 (67.4)	46 106 (58.1)
Diabetes	21 030 (24.2)	1568 (38.8)	972 (28.6)	18 490 (23.3)
Smoking status^b^^,^^c^				
Never	34 408 (42.0)	1697 (46.5)	1276 (40.5)	31 435 (41.9)
Former	29 841 (36.4)	1391 (38.1)	1219 (38.7)	27 231 (36.3)
Active	17 631 (20.3)	559 (15.3)	657 (20.8)	16 415 (21.8)
Previous				
MI	18 332 (21.1)	1834 (45.4)	842 (24.8)	15 656 (19.7)
PCI	12 387 (14.3)	1085 (26.9)	533 (15.7)	10 769 (13.6)
CABG	6094 (7.0)	678 (16.8)	270 (7.9)	5146 (6.5)
Stroke	8870 (10.2)	895 (22.2)	499 (14.7)	7476 (9.4)
Bleeding	4944 (5.7)	386 (9.6)	396 (11.7)	4162 (5.3)
Heart failure	8001 (9.2)	912 (22.6)	517 (15.2)	6572 (8.3)
Cancer	2963 (3.4)	238 (5.9)	244 (7.2)	2481 (3.1)
LEAD	4788 (5.5)	490 (12.1)	315 (9.3)	3983 (5.0)
COPD	6575 (7.6)	419 (10.4)	395 (11.6)	5761 (7.3)
Previous kidney failure	3931 (4.5)	508 (12.6)	311 (9.1)	3112 (3.9)
Invasive treatment in-hospital				
Coronary angiography	73 190 (84.4)	2398 (59.4)	2680 (78.9)	68 112 (85.9)
PCI	59 802 (69.0)	1763 (43.6)	2232 (65.7)	55 807 (70.4)
CABG	4489 (5.2)	116 (2.9)	131 (3.9)	4242 (5.3)
Laboratory variable, median (IQR)^b^				
Hemoglobin, g/dL^a^^,^^d^	13.9 (12.7-15.0)	13.1 (11.9-14.3)	13.3 (11.9-14.5)	14.0 (12.8-15.0)
Creatinine, mg/dL^e^	0.93 (0.78-1.12)	1.03 (0.83-1.37)	1.37 (0.80-1.27)	0.91 (0.77-1.11)
eGFR, mL/min	78 (59-91)	62 (43-81)	69 (49-86)	79 (60-91)
Discharge medication				
Antithrombotic therapy				
Aspirin^f^	80 772 (93.1)	3597 (89.1)	3111 (91.5)	74 298 (93.7)
Ticagrelor^f^	48 760 (56.2)	1434 (35.5)	1732 (51.0)	45 594 (57.9)
Clopidogrel^f^^,^^g^	23 682 (27.3)	1697 (42.0)	1040 (30.6)	20 944 (26.4)
Prasugrel^f^	890 (1.0)	22 (0.5)	35 (1.0)	833 (1.1)
Warfarin	7023 (8.1)	440 (10.9)	403 (11.9)	6180 (7.8)
NOAC^f^	2912 (3.4)	161 (4.0)	122 (3.6)	2630 (3.3)
SAPT	11 461 (13.2)	770 (19.1)	479 (14.1)	10 212 (12.9)
DAPT	69 217 (79.8)	2847 (70.5)	2613 (76.9)	63 757 (80.4)
Dual (SAPT+APT)	4280 (4.9)	286 (7.1)	213 (6.3)	3709 (4.7)
Triple (DAPT+APT)	3877 (4.5)	179 (4.4)	217 (6.4)	3481 (4.4)
Other medication				
β-blocker	76 369 (88.0)	3565 (88.3)	2951 (86.8)	69 853 (88.1)
Calcium channel blocker	15 555 (17.9)	1082 (26.8)	697 (20.5)	13 776 (17.4)
Digoxin	1143 (1.3)	124 (3.1)	84 (2.5)	1235 (1.6)
Diuretics	22 620 (26.1)	1882 (46.6)	1212 (35.7)	19 528 (24.6)
Statins	77 958 (89.9)	3275 (81.1)	2959 (87.1)	71 724 (90.4)

Abbreviations: APT, antiplatelet therapy; CABG, coronary artery bypass grafting; COPD, chronic obstructive pulmonary disease; DAPT, dual antiplatelet therapy; eGFR, estimated glomerular filtration rate; LEAD, lower extremity artery disease; MI, myocardial infarction; NOAC, non–vitamin K antagonist oral anticoagulant; PCI, percutaneous coronary intervention; SAPT, single antiplatelet therapy; STEMI, ST-segment elevation MI.

SI conversion: To convert creatinine to micromoles per liter, multiply by 88.4; hemoglobin to grams per liter, multiply by 10.

^a^ Continuous variables: age, hemoglobin, and creatinine were handled in restricted cubic splines in the primary and sensitivity analyses.

^b^ Smoking status and the laboratory variables were not included in the Cox proportional hazards regression models in the primary analyses and were used only in a sensitivity analysis.

^c^ Data available on 81 880 patients (94.4%).

^d^ Data available on 81 878 patients (94.4%).

^e^ Data available on 83 575 patients (96.4%).

^f^ Not included in the Cox proportional hazards regression.

^g^ Ticlopidine was included in the clopidogrel group.

When assessing the characteristics of patients by their postdischarge event status (Table 1), we noted that patients experiencing an ischemic or bleeding event compared with those who did not experience any event were older (median age: ischemic, 79 years [IQR, 70-86] years; bleeding, 76 years [IQR, 68-83 years]; neither, 70 years [IQR, 62-79 years]), had more comorbidities, including hypertension (ischemic, 3150 [78.0%]; bleeding 2290 [67.4%]; neither, 46 106 [58.1%]), previous MI (ischemic, 1834 [45.4%]; bleeding, 842 [24.8%]; neither, 15 656 [19.7%), previous stroke (ischemic, 895 [22.2%]; bleeding, 499 [14.7%]; neither, 7476 [9.4%]), previous bleeding (ischemic, 386 [9.6%]; bleeding, 396 [11.7%]; neither, 4162 [5.3%]), were more often treated with OAC (ischemic, 601 [14.9%]; bleeding: 525 [15.4%]; neither, 8810 [11.1%]), and were less often treated with DAPT (ischemic, 2847 [70.5%]; bleeding, 2613 [76.9%]; neither, 63 757 [80.4%]) and ticagrelor (ischemic, 1434 [35.5%]; bleeding, 1732 [51%] neither, 45 594 [57.5%]). Patients with ischemic events vs those with bleeding events were older (median age, 79 [IQR, 70-86] years vs 76 [IQR, 68-83] years) and had more cardiovascular risk factors, including hypertension (ischemic, 3150 [78.0%]; bleeding, 2290 [67.4%]), diabetes (ischemic, 1568 [38.1%]), bleeding (972 [28.6%]), previous MI (ischemic, 1834 [45.4%], bleeding, 842 [24.8%]), and previous stroke (ischemic, 895 [22.2%]; bleeding, 499 [14.7%]). while those with bleeding events vs ischemic events were more likely to have experienced a previous bleeding event (396 [11.7%] vs 386 [9.6%]) and were more often treated with triple therapy (217 [6.4%] vs 179 [4.4%]) and potent P2Y12 inhibitors (1767 [52%] vs 1456 [36%]).

### Incidence of Ischemic and Bleeding Events

**Figure 2.**
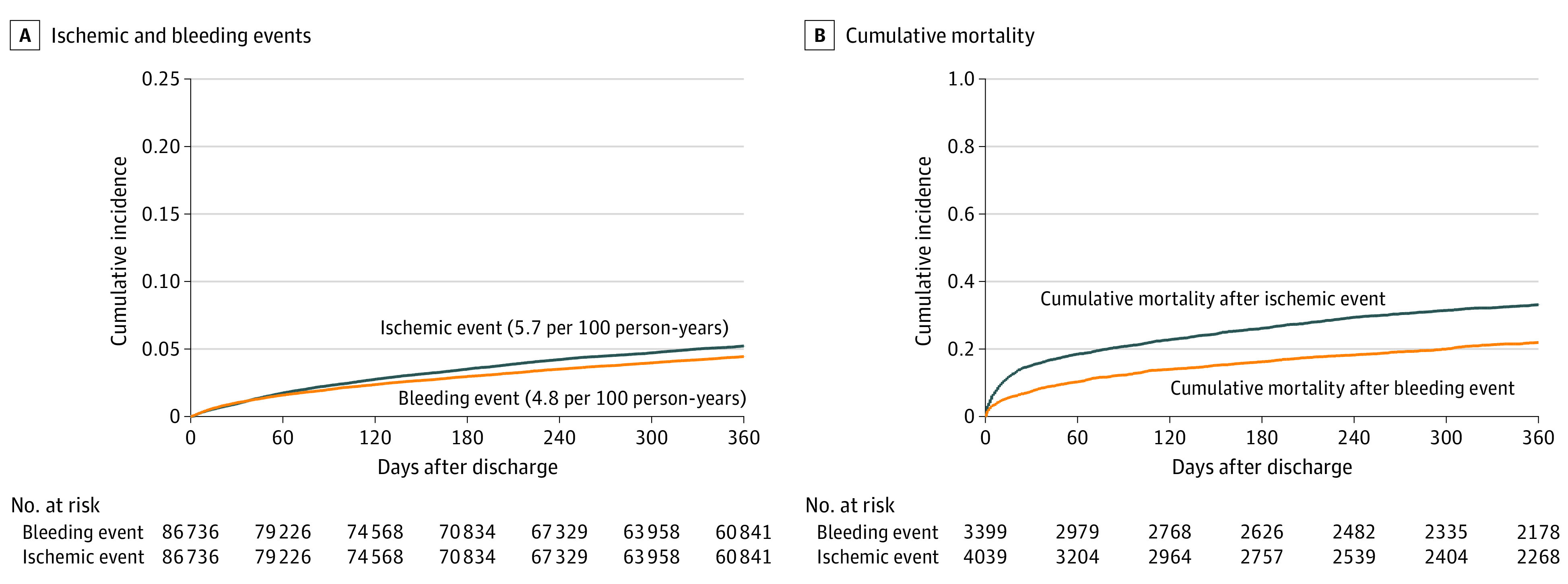
Incidence Rates of Ischemic and Bleeding Events and Mortality After Ischemic and Bleeding Events A, Incidence rates of ischemic and bleeding events during 365 days after discharge from the index myocardial infarction. B, Mortality after an ischemic event and bleeding event.

During 365 days after discharge from the index MI, 4039 patients experienced a first ischemic event (incidence rate, 5.7 events per 100 person-years), and 3399 patients experienced a first bleeding event (incidence rate, 4.8 events per 100 person-years) (Figure 2A). A total of 2863 of the ischemic events were recurrent MI and 1176 were ischemic stroke. Bleeding events by type of bleeding are reported in eTable 4 in the Supplement. The median time to the first ischemic event was 102 (IQR, 38-204) days and to the first bleeding event was 95 (IQR, 30-207) days among those experiencing such events. During the first year after discharge from the index MI, 140 patients experiencing a first ischemic event had a subsequent bleeding event and 142 of those experiencing a first bleeding event had a subsequent ischemic event.

### Primary Analyses

In the analyses comparing the risk of 1-year mortality after an ischemic event and bleeding event vs no event, 9671 patients died during follow-up. Of these, 1292 patients died after a first ischemic event (incidence rate, 46.2 deaths per 100 person-years), 715 died after a first bleeding event (incidence rate, 27.1 deaths per 100 person-years), and 7664 died after no event (incidence rate, 6.2 deaths per 100 person-years). Compared with no event, both ischemic events (aHR, 4.16; 95% CI, 3.91-4.43) and bleeding events (aHR, 3.43; 95% CI, 3.17-3.71) were associated with an increased risk of death (Table 2). In the analyses restricted to patients experiencing a first ischemic or bleeding event, an ischemic event vs a bleeding event was associated with a higher risk of death (aHR, 1.27; 95% CI, 1.15-1.40) (Table 2 and Figure 2B).

**Table 2.** Crude and Adjusted HRs for Death After an Ischemic Event and Bleeding Event Among Patients Discharged After an MI in 2012-2017

Variable	No. (%) events	Events per 100 person-years	No. deaths	Deaths per 100 person-years	HR (95% CI)			
					Event vs no event		Ischemic event vs bleeding event	
					Crude	Adjusted^a^	Crude	Adjusted^a^
No event	NA	NA	7664	6.2	1 [Reference]	1 [Reference]	NA	NA
Ischemic event	4039 (4.7)	5.7	1292	46.2	9.01 (8.48-9.58)	4.16 (3.91-4.43)	1.65 (1.51-1.82)	1.27 (1.15-1.40)
Bleeding event	3399 (3.9)	4.8	715	27.1	5.25 (4.86-5.68)	3.43 (3.17-3.71)	1 [Reference]	1 [Reference]

Abbreviations: HR, hazard ratio; NA, not applicable.

^a^ Adjusted for age (restricted cubic splines), sex, year of discharge, ST-elevation myocardial infarction, hypertension, diabetes, previous myocardial infarction, previous percutaneous coronary intervention, previous coronary artery bypass grafting, previous stroke, previous bleeding, previous heart failure, previous cancer, previous lower extremity artery disease, chronic obstructive pulmonary disease, previous kidney failure, coronary angiography, in-hospital percutaneous coronary intervention, in-hospital coronary artery bypass grafting, antithrombotic treatment strategy at discharge (single antiplatelet therapy, dual antiplatelet therapy, dual, and triple), and discharge medication (β-blocker, calcium channel blocker, digoxin, diuretics, and statins).

### Secondary Analysis

Characteristics of patients discharged in 1997-2000, 2001-2011, and 2012-2017 are reported in eTable 5 in the Supplement. During 365 days after discharge, the incidence rates of first ischemic events vs bleeding events were 11.6 events per 100 person-years vs 2.5 events per 100 person-years for those discharged in 1997-2000, 9.6 events per 100 person-years vs 3.5 events per 100 person-years for those discharged in 2001-2011, and 5.7 events per 100 person-years vs 4.8 events per 100 person-years for those discharged in 2012-2017. The aHRs for 1-year mortality after an ischemic vs bleeding event were 1.17 (95% CI, 1.02-1.35) in 1997-2000, 1.18 (95% CI, 1.11-1.27) in 2001-2011, and 1.27 (95% CI, 1.15-1.40) in 2012-2017 (Table 3). There was no significant interaction between ischemic vs bleeding event and time period (*P* values for interaction vs 1997-2000: *P* = .91 for 2001-2011 and *P* = .65 for 2012-2017).

**Table 3.** Incidence Rates of Ischemic and Bleeding Events and Adjusted HRs for Death After an Ischemic vs Bleeding Event in 3 Time Periods From 1997-2017

Time period	Events per 100 person-years		Deaths per 100 person-years		Death after ischemic vs bleeding event, adjusted HR (95% CI)^a^
	Ischemic	Bleeding	After ischemic event	After bleeding event	
1997-2000	11.6	2.5	52.3	39.5	1.17 (1.02-1.35)
2001-2011	9.6	3.5	49.5	31.8	1.18 (1.11-1.27)
2012-2017	5.7	4.8	46.2	27.1	1.27 (1.15-1.40)

Abbreviation: HR, hazard ratio.

^a^ Adjusted for age (restricted cubic splines), sex, year of discharge, ST-elevation myocardial infarction, hypertension, diabetes, previous myocardial infarction, previous percutaneous coronary intervention, previous coronary artery bypass grafting, previous stroke, previous bleeding, previous heart failure, previous cancer, previous lower extremity artery disease, chronic obstructive pulmonary disease, previous kidney failure, coronary angiography, in-hospital percutaneous coronary intervention, in-hospital coronary artery bypass grafting, antithrombotic treatment strategy at discharge (single antiplatelet therapy, dual antiplatelet therapy, dual, and triple) and discharge medication (β-blocker, calcium blocker, digoxin, diuretics, and statins).

### Sensitivity Analyses

Adjustment for smoking status, hemoglobin, and estimated glomerular filtration rate did not significantly change the estimated mortality risks. Among the 77 293 patients (89.1% of the total population included in the primary analyses) with data on these variables available, the aHR adjusted for variables in eTable 1 in the Supplement for an ischemic event vs no event was 4.20 (95% CI, 3.92-4.51) without and 4.15 (95% CI, 3.87-4.45) with adjustment for the additional variables. The corresponding aHRs for a bleeding event vs no event were 3.63 (95% CI, 3.33-3.96) and 3.36 (95% CI, 3.08-3.67). The aHRs for an ischemic vs bleeding event were 1.25 (95% CI, 1.12-1.39) without and 1.28 (95% CI, 1.15-1.43) with adjustment for the additional variables (eTable 6 in the Supplement). In analyses categorizing patients experiencing both an ischemic and a bleeding event on the same day as exposed to a bleeding event, the findings were largely similar to those of the primary analyses (eTable 7 in the Supplement).

### Post hoc Analyses

In analyses restricting the ischemic event definition to recurrent MI, the difference in the mortality risk associated with the 2 types of events was smaller than in the primary analysis (eTable 8 in the Supplement). Findings were largely similar in the 2 subgroups by OAC status at discharge (eTable 9 in the Supplement). The HR for 30-day mortality after an ischemic vs bleeding event was larger than that for 1-year mortality (eTable 10 in the Supplement).

## Discussion

In this register-based nationwide cohort study of patients in Sweden with a recent MI, recurrent ischemic events were more common and associated with a higher 1-year mortality risk compared with bleeding events. The relative mortality risk associated with an ischemic vs bleeding event had not changed significantly over the past 2 decades.

In contrast to our findings, previous studies of patients with a recent MI or PCI have found similar mortality risks associated with recurrent MI vs major bleeding event.^17^ In addition, in many of these studies, the incidence of recurrent MI was lower or similar to that of bleeding events. There are several potential explanations for the contrasting findings in our study vs previous studies. First, we used a nationwide sample of patients seen in routine clinical practice, but previous studies were predominantly performed using data from clinical trials with highly selected populations. In comparison with previous studies, the population in our study had a higher risk of both ischemic and bleeding events as well as mortality. Second, we included ischemic stroke in the definition of an ischemic event because the risk of this outcome guides the choice of, and can be modified with, antithrombotic treatment. All but 2 previous studies assessed only recurrent MI vs bleeding.^18,19,21,29,30,31,32,33,34^ The 2 studies including ischemic stroke^13,20^ were based on randomized clinical trials and 1 of the studies included only patients at low risk who were alive and free of ischemic and bleeding events at 12 months after PCI (46% MI).^7,13^ Third, we included only ischemic and bleeding events occurring after discharge from the MI because the risk of these events guides antithrombotic therapy; some previous studies^19,30,35^ have also included events during in-hospital stay for the initial MI or PCI. Taken together, by using nationwide registers to include more than 85 000 patients with MI seen in routine clinical practice with almost complete follow-up regarding hospitalizations and death, our study adds to the knowledge regarding the relative incidence of and mortality risk associated with ischemic vs bleeding events.

The mortality risk associated with a bleeding event varies substantially depending on the definition of such events.^36^ The bleeding definition used in our study included any bleeding that led to or occurred during rehospitalization; as such, the definition was broad and included both minor and major bleedings. Although it is likely that the mortality risk associated with a bleeding event would be higher in analyses restricted to severe bleedings, such an outcome definition would also lead to a lower incidence rate of bleeding. Despite the use of a broad bleeding outcome, we found that the incidence of recurrent ischemic events was approximately 20% higher than that of bleeding events.

When using risk-based strategies for guiding treatment decisions, it is necessary to not only consider the relative mortality risks but also account for the incidence of the events of interest. It may be best to guide clinical decisions by the absolute risk increase or reduction conferred by the treatment, and this estimate depends on the incidence of the event, the relative risk of the event associated with the treatment and, potentially, the association of the event with death. One way of approximating the importance of an exposure while accounting for both how common it is and its association with mortality is to calculate the population-attributable fraction.^37^ Using the aHR and incidence rates in our study population, the population-attributable fraction was 10.1% for ischemic events and 5.2% for bleeding events, indicating that ischemic events might have a larger total influence on mortality in the population. In addition, we defined ischemic and bleeding events based on diagnoses registered during hospitalizations. Given that deaths due to recurrent ischemic events that occur without earlier hospitalization (eg, at home or at nursing facilities) are likely to be more common than such deaths from bleeding events, it is possible that our study has underestimated the relative risk of death associated with an ischemic vs bleeding event.

Because data from more than a decade ago, before broad use of potent P2Y12 inhibitors^19,30,31,32,33,35,38^ and PCI^19,30,31^ may not be generalizable to contemporary practice, we assessed whether the association of ischemic vs bleeding events with mortality had changed during the past 2 decades. In accordance with a recent meta-analysis of 16 trials from 2008 to 2020,^17^ we found no significant interaction between time period and type of event with respect to the association with mortality risk. However, as has been described in previous studies,^22,23,39^ the rates of ischemic events in 2012-2017 were less than half of those in 1997-2000 and the rates of bleeding events had nearly doubled. Based on the data in our study, the population-attributable fraction for ischemic events had decreased from 16.5% to 10.1% and increased for bleeding events from 2.8% to 5.2% during the study period.

### Limitations

This study has limitations. First, because ischemic and bleeding events are not interventions, it is not possible to define a causal effect of these exposures on mortality^40^; as in previous studies, the estimates from our analyses should be considered as theoretical estimations of the relative importance of ischemic vs bleeding events with respect to mortality risk. Second, although we adjusted our analyses for many covariates, the possibility of confounding due to unmeasured patient characteristics affecting both the risk of ischemic vs bleeding events and mortality cannot be ruled out. Third, although validation studies in Swedish registers have shown high positive predictive values for cardiovascular and bleeding events,^25,27^ misclassification and underreporting of events might have introduced bias in our analyses. Fourth, we only assessed and compared the risk of mortality associated with ischemic and bleeding events although these events may also be differentially associated with reductions in quality of life and long-term comorbidity.

## Conclusions

In this nationwide study of patients with a recent MI, we assessed the association of postdischarge ischemic events and bleeding events with mortality. We found that ischemic events were more common and associated with higher mortality risk as compared with bleeding events.

## Notes

Supplement. **eMethods.** Description of Data Sources **eTable 1.** Variables Used for Adjustment of Cox Regression Models and Proportion Missing Before Patients With Missing Data Were Excluded **eTable 2.** Definition of Ischemic Events **eTable 3.** *ICD-10* Codes Used to Define Bleeding Events **eTable 4.** Number of Bleeding Events by Type of Bleeding **eTable 5.** Characteristics of Patients Discharged After an MI in 1997-2000, 2001-2012 and 2012-2017 **eTable 6.** Results of Sensitivity Analyses With and Without Adjustment for Hemoglobin, Smoking Status, and eGFR **eTable 7.** Crude and Adjusted Hazard Ratios (HRs) for Death After an Ischemic Event and Bleeding Event Among Patients Discharged After an MI in 2012-2017 in Analyses Categorizing Patients Who Experienced Both an Ischemic and Bleeding Event on the Same Day as Exposed to a Bleeding Event **eTable 8.** Crude and Adjusted Hazard Ratios (HRs) for Death After a Recurrent MI and Bleeding Event Among Patients Discharged After an MI in 2012-2017 **eTable 9.** Crude and Adjusted Hazard Ratios (HRs) for Death After an Ischemic Event and Bleeding Event Among Patients Discharged After an MI in 2012-2017 by OAC Treatment Status at Discharge **eTable 10.** Crude and Adjusted Hazard Ratios (HRs) for Death Within 30 Days After an Ischemic Event vs Bleeding Event Among Patients Discharged After an MI in 2012-2017
eReferences

## References

[1] Valgimigli M, Bueno H, Byrne RA, ; ESC Scientific Document Group; ESC Committee for Practice Guidelines (CPG); ESC National Cardiac Societies. 2017 ESC focused update on dual antiplatelet therapy in coronary artery disease developed in collaboration with EACTS: the task force for dual antiplatelet therapy in coronary artery disease of the European Society of Cardiology (ESC) and of the European Association for Cardio-Thoracic Surgery (EACTS). Eur Heart J. 2018;39(3):213-260. doi:10.1093/eurheartj/ehx419 28886622

[2] Collet JP, Thiele H, Barbato E, ; ESC Scientific Document Group. 2020 ESC Guidelines for the management of acute coronary syndromes in patients presenting without persistent ST-segment elevation. Eur Heart J. 2021;42(14):1289-1367. doi:10.1093/eurheartj/ehaa575 32860058

[3] Levine GN, Bates ER, Bittl JA, . 2016 ACC/AHA Guideline focused update on duration of dual antiplatelet therapy in patients with coronary artery disease: a report of the American College of Cardiology/American Heart Association Task Force on Clinical Practice Guidelines. J Am Coll Cardiol. 2016;68(10):1082-1115. doi:10.1016/j.jacc.2016.03.513 27036918

[4] Costa F, van Klaveren D, James S, ; PRECISE-DAPT Study Investigators. Derivation and validation of the predicting bleeding complications in patients undergoing stent implantation and subsequent dual antiplatelet therapy (PRECISE-DAPT) score: a pooled analysis of individual-patient datasets from clinical trials. Lancet. 2017;389(10073):1025-1034. doi:10.1016/S0140-6736(17)30397-5 28290994

[5] Urban P, Mehran R, Colleran R, . Defining high bleeding risk in patients undergoing percutaneous coronary intervention: a consensus document from the Academic Research Consortium for High Bleeding Risk. Eur Heart J. 2019;40(31):2632-2653. doi:10.1093/eurheartj/ehz372 31116395 PMC6736433

[6] Yeh RW, Secemsky EA, Kereiakes DJ, ; DAPT Study Investigators. Development and validation of a prediction rule for benefit and harm of dual antiplatelet therapy beyond 1 year after percutaneous coronary intervention. JAMA. 2016;315(16):1735-1749. doi:10.1001/jama.2016.3775 27022822 PMC5408574

[7] Mauri L, Kereiakes DJ, Yeh RW, ; DAPT Study Investigators. Twelve or 30 months of dual antiplatelet therapy after drug-eluting stents. N Engl J Med. 2014;371(23):2155-2166. doi:10.1056/NEJMoa1409312 25399658 PMC4481318

[8] Bonaca MP, Bhatt DL, Cohen M, ; PEGASUS-TIMI 54 Steering Committee and Investigators. Long-term use of ticagrelor in patients with prior myocardial infarction. N Engl J Med. 2015;372(19):1791-1800. doi:10.1056/NEJMoa1500857 25773268

[9] Claassens DMF, Vos GJA, Bergmeijer TO, . A genotype-guided strategy for oral P2Y12 inhibitors in primary PCI. N Engl J Med. 2019;381(17):1621-1631. doi:10.1056/NEJMoa1907096 31479209

[10] Sibbing D, Aradi D, Jacobshagen C, ; TROPICAL-ACS Investigators. Guided de-escalation of antiplatelet treatment in patients with acute coronary syndrome undergoing percutaneous coronary intervention (TROPICAL-ACS): a randomised, open-label, multicentre trial. Lancet. 2017;390(10104):1747-1757. doi:10.1016/S0140-6736(17)32155-4 28855078

[11] O’Donoghue ML, Murphy SA, Sabatine MS. the safety and efficacy of aspirin discontinuation on a background of a P2Y12 inhibitor in patients after percutaneous coronary intervention: a systematic review and meta-analysis. Circulation. 2020;142(6):538-545. doi:10.1161/CIRCULATIONAHA.120.046251 32551860

[12] Hahn JY, Song YB, Oh JH, ; SMART-DATE investigators. 6-Month versus 12-month or longer dual antiplatelet therapy after percutaneous coronary intervention in patients with acute coronary syndrome (SMART-DATE): a randomised, open-label, non-inferiority trial. Lancet. 2018;391(10127):1274-1284. doi:10.1016/S0140-6736(18)30493-8 29544699

[13] Secemsky EA, Yeh RW, Kereiakes DJ, ; Dual Antiplatelet Therapy (DAPT) Study Investigators. Mortality following cardiovascular and bleeding events occurring beyond 1 year after coronary stenting: a secondary analysis of the Dual Antiplatelet Therapy (DAPT) Study. JAMA Cardiol. 2017;2(5):478-487. doi:10.1001/jamacardio.2017.0063 28297015 PMC5814981

[14] Kim BK, Hong SJ, Cho YH, ; TICO Investigators. Effect of ticagrelor monotherapy vs ticagrelor with aspirin on major bleeding and cardiovascular events in patients with acute coronary syndrome: the TICO randomized clinical trial. JAMA. 2020;323(23):2407-2416. doi:10.1001/jama.2020.7580 32543684 PMC7298605

[15] Ndrepepa G, Berger PB, Mehilli J, . Periprocedural bleeding and 1-year outcome after percutaneous coronary interventions: appropriateness of including bleeding as a component of a quadruple end point. J Am Coll Cardiol. 2008;51(7):690-697. doi:10.1016/j.jacc.2007.10.040 18279731

[16] Valgimigli M, Frigoli E, Heg D, ; MASTER DAPT Investigators. Dual antiplatelet therapy after PCI in patients at high bleeding risk. N Engl J Med. 2021;385(18):1643-1655. doi:10.1056/NEJMoa2108749 34449185

[17] Piccolo R, Oliva A, Avvedimento M, . Mortality after bleeding versus myocardial infarction in coronary artery disease: a systematic review and meta-analysis. EuroIntervention. 2021;17(7):550-560. doi:10.4244/EIJ-D-20-01197 33840639 PMC9725060

[18] Valgimigli M, Costa F, Lokhnygina Y, . Trade-off of myocardial infarction vs. bleeding types on mortality after acute coronary syndrome: lessons from the Thrombin Receptor Antagonist for Clinical Event Reduction in Acute Coronary Syndrome (TRACER) randomized trial. Eur Heart J. 2017;38(11):804-810. 28363222 10.1093/eurheartj/ehw525 PMC5837470

[19] Mehran R, Pocock SJ, Stone GW, . Associations of major bleeding and myocardial infarction with the incidence and timing of mortality in patients presenting with non–ST-elevation acute coronary syndromes: a risk model from the ACUITY trial. Eur Heart J. 2009;30(12):1457-1466. doi:10.1093/eurheartj/ehp110 19351691 PMC2695951

[20] Ducrocq G, Schulte PJ, Budaj A, . Balancing the risk of spontaneous ischemic and major bleeding events in acute coronary syndromes. Am Heart J. 2017;186:91-99. doi:10.1016/j.ahj.2017.01.010 28454837

[21] Hara H, Takahashi K, Kogame N, . Impact of bleeding and myocardial infarction on mortality in all-comer patients undergoing percutaneous coronary intervention. Circ Cardiovasc Interv. 2020;13(9):e009177. doi:10.1161/CIRCINTERVENTIONS.120.009177 32838554

[22] Szummer K, Wallentin L, Lindhagen L, . Improved outcomes in patients with ST-elevation myocardial infarction during the last 20 years are related to implementation of evidence-based treatments: experiences from the SWEDEHEART registry 1995-2014. Eur Heart J. 2017;38(41):3056-3065. doi:10.1093/eurheartj/ehx515 29020314 PMC5837507

[23] Szummer K, Wallentin L, Lindhagen L, . Relations between implementation of new treatments and improved outcomes in patients with non–ST-elevation myocardial infarction during the last 20 years: experiences from SWEDEHEART registry 1995 to 2014. Eur Heart J. 2018;39(42):3766-3776. doi:10.1093/eurheartj/ehy554 30239671

[24] Jernberg T, Attebring MF, Hambraeus K, . The Swedish web-system for enhancement and development of evidence-based care in heart disease evaluated according to recommended therapies (SWEDEHEART). Heart. 2010;96(20):1617-1621. doi:10.1136/hrt.2010.198804 20801780

[25] Ludvigsson JF, Andersson E, Ekbom A, . External review and validation of the Swedish national inpatient register. BMC Public Health. 2011;11:450. doi:10.1186/1471-2458-11-450 21658213 PMC3142234

[26] Ludvigsson JF, Almqvist C, Bonamy AK, . Registers of the Swedish total population and their use in medical research. Eur J Epidemiol. 2016;31(2):125-136. doi:10.1007/s10654-016-0117-y 26769609

[27] Friberg L, Skeppholm M. Usefulness of health registers for detection of bleeding events in outcome studies. Thromb Haemost. 2016;116(6):1131-1139. 27617328 10.1160/TH16-05-0400

[28] Therneau T, Crowson C, Atkinson E. Using time dependent covariates and time dependent coefficients in the Cox model. March 2, 2022. Accessed July 22, 2022. https://cran.r-project.org/web/packages/survival/vignettes/timedep.pdf

[29] Montalescot G, Gallo R, White HD, ; STEEPLE Investigators. Enoxaparin versus unfractionated heparin in elective percutaneous coronary intervention 1-year results from the STEEPLE (Safety and Efficacy of Enoxaparin in Percutaneous Coronary Intervention Patients, an international randomized evaluation) trial. JACC Cardiovasc Interv. 2009;2(11):1083-1091. doi:10.1016/j.jcin.2009.08.016 19926048

[30] Kim YH, Lee JY, Ahn JM, . Impact of bleeding on subsequent early and late mortality after drug-eluting stent implantation. JACC Cardiovasc Interv. 2011;4(4):423-431. doi:10.1016/j.jcin.2010.12.008 21511222

[31] Kikkert WJ, Zwinderman AH, Vis MM, . Timing of mortality after severe bleeding and recurrent myocardial infarction in patients with ST-segment-elevation myocardial infarction. Circ Cardiovasc Interv. 2013;6(4):391-398. doi:10.1161/CIRCINTERVENTIONS.113.000425 23941861

[32] Kazi DS, Leong TK, Chang TI, Solomon MD, Hlatky MA, Go AS. Association of spontaneous bleeding and myocardial infarction with long-term mortality after percutaneous coronary intervention. J Am Coll Cardiol. 2015;65(14):1411-1420. doi:10.1016/j.jacc.2015.01.047 25857906

[33] Généreux P, Giustino G, Witzenbichler B, . Incidence, predictors, and impact of post-discharge bleeding after percutaneous coronary intervention. J Am Coll Cardiol. 2015;66(9):1036-1045. doi:10.1016/j.jacc.2015.06.1323 26314532

[34] Caneiro-Queija B, Abu-Assi E, Raposeiras-Roubín S, . Differential prognostic impact on mortality of myocardial infarction compared with bleeding severity in contemporary acute coronary syndrome patients. Rev Esp Cardiol (Engl Ed). 2018;71(10):829-836. doi:10.1016/j.recesp.2018.02.005 29656987

[35] Baber U, Dangas G, Chandrasekhar J, . Time-dependent associations between actionable bleeding, coronary thrombotic events, and mortality following percutaneous coronary intervention: results From the PARIS Registry. JACC Cardiovasc Interv. 2016;9(13):1349-1357. doi:10.1016/j.jcin.2016.04.009 27388822

[36] Vranckx P, White HD, Huang Z, . Validation of BARC bleeding criteria in patients with acute coronary syndromes: the TRACER trial. J Am Coll Cardiol. 2016;67(18):2135-2144. doi:10.1016/j.jacc.2016.02.056 27151345

[37] Valgimigli M, Gargiulo G. Deciding on the duration of dual antiplatelet therapy—when the choice between 2 evils is still evil. JAMA Cardiol. 2017;2(5):488-489. doi:10.1001/jamacardio.2017.0125 28297004

[38] Stone GW, Clayton T, Deliargyris EN, Prats J, Mehran R, Pocock SJ. Reduction in cardiac mortality with bivalirudin in patients with and without major bleeding: the HORIZONS-AMI trial (Harmonizing Outcomes with Revascularization and Stents in Acute Myocardial Infarction). J Am Coll Cardiol. 2014;63(1):15-20. doi:10.1016/j.jacc.2013.09.027 24140664

[39] Simonsson M, Wallentin L, Alfredsson J, . Temporal trends in bleeding events in acute myocardial infarction: insights from the SWEDEHEART registry. Eur Heart J. 2020;41(7):833-843. doi:10.1093/eurheartj/ehz593 31504404

[40] Hernán MA, Taubman SL. Does obesity shorten life? the importance of well-defined interventions to answer causal questions. Int J Obes (Lond). 2008;32(suppl 3):S8-S14. doi:10.1038/ijo.2008.82 18695657

