# Supplementary material for: Association of Ischemic and Bleeding Events With Mortality Among Patients in Sweden With Recent Acute Myocardial Infarction Receiving Antithrombotic Therapy
Source: JAMA Netw Open. 2022 Aug 29;5(8):e2220030. doi: 10.1001/jamanetworkopen.2022.20030 (PMC9425148; doi:10.1001/jamanetworkopen.2022.20030)
Supplement: Supplement. — eMethods. Description of Data Sources eTable 1. Variables Used for Adjustment of Cox Regression Models and Proportion Missing Before Patients With Missing Data Were Excluded eTable 2. Definition of Ischemic Events eTable 3. ICD-10 Codes Used to Define Bleeding Events eTable 4. Number of Bleeding Events by Type of Bleeding eTable 5. Characteristics of Patients Discharged After an MI in 1997-2000, 2001-2012 and 2012-2017 eTable 6. Results of Sensitivity Analyses With and Without Adjustment for Hemoglobin, Smoking Status, and eGFR eTable 7. Crude and Adjusted Hazard Ratios (HRs) for Death After an Ischemic Event and Bleeding Event Among Patients Discharged After an MI in 2012-2017 in Analyses Categorizing Patients Who Experienced Both an Ischemic and Bleeding Event on the Same Day as Exposed to a Bleeding Event eTable 8. Crude and Adjusted Hazard Ratios (HRs) for Death After a Recurrent MI and Bleeding Event Among Patients Discharged After an MI in 2012-2017 eTable 9. Crude and Adjusted Hazard Ratios (HRs) for Death After an Ischemic Event and Bleeding Event Among Patients Discharged After an MI in 2012-2017 by OAC Treatment Status at Discharge eTable 10. Crude and Adjusted Hazard Ratios (HRs) for Death Within 30 Days After an Ischemic Event vs Bleeding Event Among Patients Discharged After an MI in 2012-2017 eReferences [file jamanetwopen-e2220030-s001.pdf]

Supplementary Online Content

Simonsson M, Alfredsson J, Szummer K, Jernberg T, Ueda P. Association of ischemic and bleeding events with mortality among patients in Sweden with recent acute myocardial infarction receiving antithrombotic therapy. *JAMA Netw Open*. 2022;5(8): e2220030. doi:10.1001/jamanetworkopen.2022.20030

eMethods. Description of Data Sources

eTable 1. Variables Used for Adjustment of Cox Regression Models and Proportion Missing Before Patients With Missing Data Were Excluded

eTable 2. Definition of Ischemic Events

eTable 3. *ICD-10* Codes Used to Define Bleeding Events

eTable 4. Number of Bleeding Events by Type of Bleeding

eTable 5. Characteristics of Patients Discharged After an MI in 1997-2000, 2001-2012 and 2012-2017

eTable 6. Results of Sensitivity Analyses With and Without Adjustment for Hemoglobin, Smoking Status, and eGFR

eTable 7. Crude and Adjusted Hazard Ratios (HRs) for Death After an Ischemic Event and Bleeding Event Among Patients Discharged After an MI in 2012-2017 in Analyses Categorizing Patients Who Experienced Both an Ischemic and Bleeding Event on the Same Day as Exposed to a Bleeding Event

eTable 8. Crude and Adjusted Hazard Ratios (HRs) for Death After a Recurrent MI and Bleeding Event Among Patients Discharged After an MI in 2012-2017

eTable 9. Crude and Adjusted Hazard Ratios (HRs) for Death After an Ischemic Event and Bleeding Event Among Patients Discharged After an MI in 2012-2017 by OAC Treatment Status at Discharge

eTable 10. Crude and Adjusted Hazard Ratios (HRs) for Death Within 30 Days After an Ischemic Event vs Bleeding Event Among Patients Discharged After an MI in 2012-2017

eReferences

This supplementary material has been provided by the authors to give readers additional information about their work.

© 2022 Simonsson M et al. *JAMA Network Open.*

eMethods. Description of data sources

The SWEDEHEART (Swedish Web-system for Enhancement and Development of Evidence-based care in Heart disease Evaluated According to Recommended Therapies)[1] registry collects information on baseline characteristics, in-hospital events and treatment and medications used on arrival, in-hospital and at discharge from all Swedish coronary care units (n=72). The diagnosis of myocardial infarction (MI) is determined by the physician responsible for treating the patient. The registry is monitored regularly, showing a 95-96% agreement between key variables in the registry and electronic health records. The National Patient Register (NPR)[2] includes all International Classification of Diseases (ICD) codes for all hospital admissions since 1987 and outpatient specialist care visits since 2001 but does not cover primary care visits. Since 1997 the NPR uses the tenth version, ICD-10. In our analysis, only hospital admissions were used to capture outcome events. The Swedish Population Registers[3] hold information on major life events such as birth, death, marital status with close to full coverage for all births and deaths in Sweden. In our analyses, emigration was not accounted for.

© 2022 Simonsson M et al. *JAMA Network Open.*

eTable 1: Variables used for adjustment of Cox regression models and proportion missing before patients with missing data were excluded. N = 86 873 of patients discharged with antithrombotic therapy after a myocardial infarction in 2012-2017.

**Variable**	**Data source and definition**	**N (%) missing**
**Demographics**		
Female sex	Swedeheart	0
Age	Swedeheart	0
STEMI	Swedeheart	<10 (<0.1)
Year of discharge	Swedeheart	0
**Medical history**		
Hypertension	Swedeheart<br>NPR (ICD-10: I10. ICD-9: 401 - 405)	0
Diabetes mellitus	Swedeheart<br>NPR (ICD-10: E10, E11, E12, E13, E14. ICD-9: 250)	0
Previous MI	Swedeheart<br>NPR (ICD-10: I21, I22, I23. ICD-9: 410, 412)	0
Previous PCI	Swedeheart<br>NPR (ICD-10: FNG00 , FNG01, FNG02, FNG03, FNG04, FNG05, FNG06. ICD-9: 3080)	0
Previous CABG	Swedeheart<br>NPR (ICD-10: (FNA, FNF, FNH, FNW. ICD-9: 3065, 3066, 3068, 3092, 3105, 3127, 3158)	0
Previous stroke	Swedeheart<br>NPR (ICD-10: I60, I61, I62, I63, I64. ICD-9: 430, 431, 432, 433, 434, 435, 436)	0
Previous bleeding	Swedeheart<br>NPR (ICD-10: I60, I61, I62 D629, D500, H356, H431, H450, H922, I850, K226, K250, K252, K254, K256, K260, K262, K264, K266, K270, K272, K274, K276, K280, K282, K284, K286, K290, K625, K920, K921, K922, N421, N938, N939, N950, R041, R042, R048, R049, R210, R319, T810, N501A. ICD-9: 430, 431, 432, 578, 285B, 456A, 459A, 530H, 531A, 531C, 531E, 531G, 532A, 532C, 532E, 532G,	0

© 2022 Simonsson M et al. *JAMA Network Open.*

	533A, 533C, 533E, 533G, 534A, 534C, 534E, 534G, 569D, 599H, 602B, 627B, 784W, 786D, 998B)	
Previous HF	Swedeheart<br>NPR (ICD-10: I50, I11. ICD-9: 428)	0
Previous cancer	Swedeheart<br>NPR (ICD-10: C14 - C20)	0
Previous LEAD	Swedeheart<br>NPR (ICD-10: I70, I71, I72, I73. ICD-9: 440, 441, 442, 443)	0
COPD	Swedeheart<br>NPR (ICD-10: J40, J41, J42, J43, J44, J45, J46, J47. ICD-9: 491, 492, 493, 494, 495, 496)	0
Previous renal failure	Swedeheart<br>NPR (ICD-10: N17, N18, N19)	0
**Invasive procedures during admission for index MI**		
Angio	Swedeheart	0
PCI	Swedeheart	0
CABG	Swedeheart	0
**Antithrombotic therapy at discharge**		
SAPT	Swedeheart	44 (<0.1)
DAPT	Swedeheart	50 (<0.1)
Dual (DAPT+OAC)	Swedeheart	28 (<0.1)
Triple (SAPT + OAC)	Swedeheart	51 (<0.1)
**Other discharge medications**		
Betablocker	Swedeheart	18 (<0.1)
Calciumblocker	Swedeheart	25 (<0.1)
Digoxin	Swedeheart	16 (<0.1)
Diuretic	Swedeheart	21 (<0.1)
Statins	Swedeheart	22 (<0.1)

Covariates were selected based on previous knowledge on risk factors for ischemic and bleeding event. None of the variables were time-updated.[4-8]

CABG: coronary artery bypass grafting, C: continuous, COPD: chronic obstructive pulmonary disease, D: dichotomous, DAPT: dual antiplatelet therapy, DM: diabetes mellitus, eGFR: estimated glomerular filtration rate, HF: heart failure, LEAD: lower extremity artery disease MI: myocardial infarction, NOAC: non-vitamin K antagonist oral anticoagulant, OAC: oral anticoagulant, PCI: percutaneous coronary intervention, SAPT: single antiplatelet therapy, STEMI: ST-segment elevation myocardial infarction

© 2022 Simonsson M et al. *JAMA Network Open.*

eTable 2 Definition of ischemic events

	**Event (occurring within 365 days from discharge after the index MI)**
MI	Readmission for MI registered in the SWEDEHEART registry (day 2-30 after discharge) Readmission registered (day 31-365 after discharge) in the NPR with ICD10: I21 as primary diagnosis.
Ischemic stroke	Readmission (day 1-365 after discharge) registered in the National Patient Register with ICD10 I63 as primary or secondary diagnosis.

ICD 10: International Statistical Classification of Diseases and Related Health Problems - Tenth Revision, MI: myocardial infarction, NPR: National Patient Register, SWEDEHEART: Swedish Web-system for Enhancement and Development of Evidence-based care in Heart disease Evaluated According to Recommended Therapies

We did not include MI events registered in SWEDEHEART during the first 2 days after discharge from the index MI and MI events registered in NPR within 30 days after discharge after the index MI. These event definitions were chosen to lower the risk of counting events related to the index MI that do not constitute a new event. Specifically, in NPR, diagnoses of MI may be registered on several occasions during the patient’s hospitalization for their index MI. This may occur when a patient is transferred from the cardiology clinic to a rehabilitation clinic or when the patient is rehospitalized shortly after discharge even if the rehospitalization is not due to a new MI event.

In SWEDEHEART, the risk of such multiple entries of the same MI is much lower. However, it may happen that the same MI is registered twice if the patient is moved between cardiology clinics during the acute phase of the MI. Therefore, we did not consider new MI diagnoses registered in SWEDEHEART during the first 2 days after discharge from the index MI.

© 2022 Simonsson M et al. *JAMA Network Open.*

eTable 3 *ICD-10* codes used to define bleeding events.

ICD-10 code	Diagnosis	Type of bleeding
I60	Subarachnoidal bleeding	Intracranial
I61	Intracerebral bleeding	Intracranial
I62	Other intracranial bleeding	Intracranial
D629	Anemi after acute larger bleeding	Other bleeding
D500	Iron deficiency anemia secondary to chronic blood loss	Other bleeding
H356	Retinal bleeding	Other bleeding
H431	Vitreous bleeding	Other bleeding
H450	Vitreous bleeding	Other bleeding
H922	Bleeding from the ear	Other bleeding
I850	Esophageal varices with bleeding	GI-bleeding
K226	Gastro-esophageal ulcer with bleeding, Mallory Weiss	GI-bleeding
K250	Ulcus ventriculi with bleeding	GI-bleeding
K252	Ulcus ventriculi with bleeding	GI-bleeding
K254	Ulcus ventriculi with bleeding	GI-bleeding
K256	Ulcus ventriculi with bleeding	GI-bleeding
K260	Ulcus duodeni with bleeding	GI-bleeding
K262	Ulcus duodeni with bleeding	GI-bleeding
K264	Ulcus duodeni with bleeding	GI-bleeding
K266	Ulcus duodeni with bleeding	GI-bleeding
K270	Ulcus ventriculi or duodeni with bleeding	GI-bleeding
K272	Ulcus ventriculi or duodeni with bleeding	GI-bleeding
K274	Ulcus ventriculi or duodeni with bleeding	GI-bleeding
K276	Ulcus ventriculi or duodeni with bleeding	GI-bleeding
K280	Reccurence of bleeding ulcer after op	GI-bleeding
K282	Reccurence of bleeding ulcer after op	GI-bleeding
K284	Reccurence of bleeding ulcer after op	GI-bleeding
K286	Reccurence of bleeding ulcer after op	GI-bleeding
K290	Acute hemorrhagic gastritis	GI-bleeding
K625	Bleeding in anus or rectum	GI-bleeding
K920	GI-bleeding	GI-bleeding
K921	GI-bleeding	GI-bleeding
K922	GI-bleeding	GI-bleeding
N421	Prostate bleeding	Urogenital bleeding
N938	Bleeding from uterus or vagina	Urogenital bleeding
N939	Bleeding from uterus or vagina	Urogenital bleeding
N950	Bleeding after menopause	Urogenital bleeding
R041	Bleeding from pharynx	Other bleeding
R042	Hemoptysis	Other bleeding
R048	Bleeding from the airways	Other bleeding
R049	Bleeding from the airways	Other bleeding
R319	Hematuria	Urogenital bleeding
T810	Bleeding complicating a procedure	Other bleeding
N501A	Bleeding from male genitalia	Urogenital bleeding

© 2022 Simonsson M et al. *JAMA Network Open.*

ICD 10: International Statistical Classification of Diseases and Related Health Problems - Tenth Revision, GI: gastrointestinal

© 2022 Simonsson M et al. *JAMA Network Open.*

eTable 4 Number of bleeding events by type of bleeding

Type of bleeding	N (%)
Intracranial bleeding[a]	286 (8.4)
GI bleeding[a]	1620 (46.7)
Urogenital bleeding[a]	652 (19.2)
Other bleeding	841 (24.7)
All bleeding	3 399 (100)

[a] One patient with both intracranial and GI bleeding was categorized as having an intracranial bleeding. Eight patients had both GI bleeding and urogenital bleeding and were categorized as having GI bleeding.

GI: gastrointestinal

© 2022 Simonsson M et al. *JAMA Network Open.*

eTable 5. Characteristics of patients discharged after an MI in 1997-2000, 2001-2012 and 2012-2017

	Year of discharge from MI		
	**1997–2000**	**2001–2011**	**2012–2017**
	N = 36 740	N= 150 393	N= 86 763
**Demographics n (%)**			
Age median years (IQR)	72 (62–79)	72 (62–80)	71 (62–80)
Female sex	12 683 (34.5)	53 716 (35.7)	29 449 (34.0)
STEMI	14 969 (40.7)	50 490 (33.6)	29 291 (33.8)
**Medical history n (%)**			
Hypertension	13 338 (36.3)	72 828 (48.4)	51 546 (59.4)
Diabetes mellitus	7 556 (20.6)	33 779 (22.4)	21 030 (24.3)
Previous MI	10 805 (29.4)	37 358 (24.8)	18 332 21.1)
Previous PCI	1 569 (4.3)	13 561 (9.0)	12 387 (14.3)
Previous CABG	1 958 (5.3)	11 049 (7.4)	6 094 (7.0
Previous stroke	3 716 (0.1)	17 623 (11.7)	8 870 (10.2)
Previous bleeding	1 345 (3.7)	7 745 (5.2)	4 944 (5.7)
Previous HF	4 434 (12.1)	17 624 (11.7)	8 001 (9.2)
Previous cancer	532 (1.5)	3 551 (2.4)	2 963 (3.4)
Previous LEAD	1 886 (5.1)	8 142 (5.4)	4 788 (5.5)
COPD	1 593 (4.3)	9 713 (6.5)	6 575 (7.6)
Previous Renal failure	552 (1.5)	4 076 (2.7)	3 931 (4.5)
**Invasive treatment in-hospital n (%)**			
Coronary angiography	3 653 (9.9)	92 643 (61.6)	73 190 (84.4)
PCI	3 794 (10.3)	70 168 (46.7)	59 802 (69.0)
CABG	572 (1.6)	4 513 (3.0)	4 489 (5.2)
**Discharge medication n (%)**			
Aspirin	33 688 (91.7)	140 565 (93.4)	80 772 (93.1)
Ticagrelor	0	27 (<0.1)	48 760 (56.2)
Clopidogrel*	2 284 (6.2)	95 261 (63.3)	23 682 (27.3)
Prasugrel	0	1 135 (0.8)	890 (1.0)
Warfarin	4 151 (11.3)	10 969 (7.3)	7 023 (8.1)
NOAK	0	13 (<0.1)	2 912 (3.4)
SAPT	30 879 (84.1)	50 315 (33.5)	11 461 (13.2)
DAPT	1 764 (4.8)	91 281 (60.7)	69 217 (79.8)
Dual (SAPT+OAC)	1 565 (4.3)	4 111 (2.7)	4 280 (4.9)
Triple (DAPT+OAC)	54 (0.2)	2 185 (1.5)	3 877 (4.5)
**Other medication**			
Beta blocker	30 114 (82.0)	133 452 (88.7)	76 369 (88.1)
Calcium blocker	5 063 (13.8)	21 264 (14.1)	15 555 (17.9)

© 2022 Simonsson M et al. *JAMA Network Open.*

Digoxin	3 785 (10.3)	6 978 (4.6)	1 143 (1.7)
Diuretics	15 616 (42.5)	54 609 (36.3)	22 620 (26.1)
Statins	14 125 (38.5)	115 585 (76.9)	77 958 (89.9)

ACEi: angiotensin-converting enzyme inhibitor, ARB: angiotensin receptor blocker, CABG: coronary artery bypass grafting, COPD: chronic obstructive pulmonary disease, DAPT: dual antiplatelet therapy, DM: diabetes mellitus, eGFR: estimated glomerular filtration rate, HF: heart failure, IQR: interquartile range, LEAD: lower extremity artery disease MI: myocardial infarction, NOAC: non-vitamin K antagonist oral anticoagulant, OAC: oral anticoagulant, PCI: percutaneous coronary intervention, SAPT: single antiplatelet therapy, STEMI: ST-segment elevation myocardial infarction

*Ticlopidine was included in the variable for clopidogrel

© 2022 Simonsson M et al. *JAMA Network Open.*

eTable 6. Results of sensitivity analyses with and without adjustment for hemoglobin, smoking status, and eGFR

		Event vs no event: Adjusted HR* for mortality (95% CI)	Ischemic vs bleeding event: Adjusted HR** for mortality (95% CI)
Ischemic event	Primary analysis	4.16 (3.91 – 4.43)	1.27 (1.15 – 1.40)
	Sensitivity analysis[a] w/o additional adjustment	4.20 (3.92 – 4.51)	1.25 (1.12 – 1.39)
	Sensitivity analysis[a] with additional adjustment***	4.15 (3.87 – 4.45)	1.28 (1.15 – 1.43)
Bleeding event	Primary analysis	3.43 (3.17 – 3.71)	reference
	Sensitivity analysis[a] w/o additional adjustment	3.63 (3.33 – 3.96)	reference
	Sensitivity analysis[a] with additional adjustment***	3.36 (3.08 – 3.67)	reference

eGFR: estimated glomerular filtration rate, HR: hazard ratio, HR: hazard ratio

***** Adjusted for age, sex, year of discharge, STEMI, hypertension, diabetes, previous MI, previous PCI, previous CABG, previous stroke, previous bleeding, previous HF, previous cancer, previous LEAD, COPD, previous renal failure, coronary angiography, in-hospital PCI , in-hospital CABG, antithrombotic treatment strategy at discharge (SAPT, DAPT, dual, triple) and discharge medication (beta blocker, calcium blocker, digoxin, diuretics and statins).

** Adjusted for the same covariates listed above plus time from discharge to the event in days.

***Additional adjustment for smoking status, hemoglobin and estimated glomerular filtration rate.

[a:] In the sensitivity analysis 77 293 (89.1% of the full cohort) patients were included; patients with missing data on hemoglobin, smoking status or creatinine were excluded (n=9 443 (10.9%)).

© 2022 Simonsson M et al. *JAMA Network Open.*

eTable 7. Crude and adjusted hazard ratios (HRs) for death after an ischemic event and bleeding event among patients discharged after an MI in 2012-2017 in analyses categorizing patients who experienced both an ischemic and bleeding event on the same day[a] as exposed to a bleeding event

					Event vs no event		Ischemic event vs bleeding event	
	N (%) events	Events per 100 person-years	N deaths	Deaths per 100 person-years	Crude HR (95% CI)	Adjusted HR (95% CI)*	Crude HR (95% CI)	Adjusted HR (95% CI) **
No event	-	-	7 664	6.2	Ref.	Ref.	N/A	N/A
Ischemic event	3 880 (4.5%)	5.4	1 225	45.3	8.86 (8.33 – 9.43)	4.06 (3.81 – 4.34)	1.54 (1.41 – 1.69)	HR 1.19 (1.08 – 1.30)
Bleeding event	3 558 (4.1%)	5.0	782	28.6	5.53 (5.13 – 5.96)	3.58 (3.32 – 3.87)	Ref.	Ref.

***** Adjusted for age, sex, year of discharge, STEMI, hypertension, diabetes, previous MI, previous PCI, previous CABG, previous stroke, previous bleeding, previous HF, previous cancer, previous LEAD, COPD, previous renal failure, coronary angiography, in-hospital PCI , in-hospital CABG, antithrombotic treatment strategy at discharge (SAPT, DAPT, dual, triple) and discharge medication (beta blocker, calcium blocker, digoxin, diuretics and statins).
** Adjusted for the same covariates listed above plus time from discharge to the event in days.

HR: hazard ratio, MI: myocardial infarction

[a]159 patients experienced an ischemic and a bleeding event on the same day.

© 2022 Simonsson M et al. *JAMA Network Open.*

eTable 8. Crude and adjusted hazard ratios (HRs) for death after a recurrent MI and bleeding event among patients discharged after an MI in 2012-2017

					Event vs no event		Ischemic event vs bleeding event	
	N (%) events	Events per 100 person-years	N deaths total n = 9 811	Deaths per 100 person-years	Crude HR (95% CI)	Adjusted HR (95% CI)*	Crude HR (95% CI)	Adjusted HR (95% CI) **
No event	-	-	8 158	6.5	Ref.	Ref.	N/A	N/A
MI event	2 907 (3.4%)	4.0	895	43.7	8.03 (7.48 – 8.62)	3.54 (3.29 – 3.80)	1.52 (1.37 – 1.67)	1.12 (1.01 – 1.25)
Bleeding event	3 489 (4.0%)	4.9	758	28.1	5.11 (4.74 – 5.51)	3.35 (3.11 – 3.62)	Ref.	Ref.

***** Adjusted for age, sex, year of discharge, STEMI, hypertension, diabetes, previous MI, previous PCI, previous CABG, previous stroke, previous bleeding, previous HF, previous cancer, previous LEAD, COPD, previous renal failure, coronary angiography, in-hospital PCI , in-hospital CABG, antithrombotic treatment strategy at discharge (SAPT, DAPT, dual, triple) and discharge medication (beta blocker, calcium blocker, digoxin, diuretics and statins).

** Adjusted for the same covariates listed above plus time from discharge to the event in days.

HR: hazard ratio, MI: myocardial infarction

© 2022 Simonsson M et al. *JAMA Network Open.*

eTable 9. Crude and adjusted hazard ratios (HRs) for death after an ischemic event and bleeding event among patients discharged after an MI in 2012-2017 by OAC treatment status at discharge

	Event n (%)	Events per 100 person-years	N deaths	Deaths per 100 person-years	Event vs no event		Ischemic event vs bleeding event	
**OAC treatment** (n=9 935)					Crude HR (95% CI)	Adjusted HR (95% CI)*	Crude HR (95% CI)	Adjusted HR (95% CI) **
No event	-	-	1 364	11.3	Ref.	Ref.	-	-
Ischemic event	601 (6.0)	8.0	221	54.8	6.38 (5.51 – 7.38)	4.40 (3.79 – 5.12)	1.48 (1.20 – 1.83)	1.32 (1.05 – 1.66)
Bleeding event	524 (5.2)	7.0	140	37.1	4.00 (3.35 – 4.77)	3.33 (2.78 – 3.98)	Ref.	Ref.
**No OAC treatment** (n=76 810)								
No event	-	-	6 300	5.7				
Ischemic event	3 438 (4.5)	5.3	1071	43.8	9.48 (8.87 – 10.13)	4.11 (3.84 – 4.40)	1.70 (1.53 – 1.88)	1.26 (1.13 – 1.40)
Bleeding event	2 875 (3.7)	4.5	575	25.4	5.41 (4.96 – 5.91)	3.44 (3.14 – 3.75)	Ref.	Ref.

***** Adjusted for age, sex, year of discharge, STEMI, hypertension, diabetes, previous MI, previous PCI, previous CABG, previous stroke, previous bleeding, previous HF, previous cancer, previous LEAD, COPD, previous renal failure, coronary angiography, in-hospital PCI , in-hospital CABG, antithrombotic treatment strategy at discharge (SAPT, DAPT, dual, triple) and discharge medication (beta blocker, calcium blocker, digoxin, diuretics and statins).
** Adjusted for the same covariates listed above plus time from discharge to the event in days.

HR: hazard ratio, MI: myocardial infarction, OAC oral anticoagulant

© 2022 Simonsson M et al. *JAMA Network Open.*

eTable 10 Crude and adjusted hazard ratios (HRs) for death within 30 days after an ischemic event vs bleeding event among patients discharged after an MI in 2012-2017

			Ischemic event vs bleeding event	
	N deaths	Deaths per 100 person-years	Crude HR (95% CI)	Adjusted HR (95% CI) *
Ischemic event N=4 039	612	207.3	2.10 (1.81 – 2.42)	1.62 (1.38 – 1.89)
Bleeding event N=3 399	256	96.9	Ref.	Ref.

***** Adjusted for age, sex, year of discharge, STEMI, hypertension, diabetes, previous MI, previous PCI, previous CABG, previous stroke, previous bleeding, previous HF, previous cancer, previous LEAD, COPD, previous renal failure, coronary angiography, in-hospital PCI , in-hospital CABG, antithrombotic treatment strategy at discharge (SAPT, DAPT, dual, triple) and discharge medication (beta blocker, calcium blocker, digoxin, diuretics and statins) and time from discharge to the event in days.

© 2022 Simonsson M et al. *JAMA Network Open.*

eReferences

1. Jernberg T, Attebring MF, Hambraeus K, et al. The Swedish Web-system for enhancement and development of evidence-based care in heart disease evaluated according to recommended therapies (SWEDEHEART). *Heart*. Oct 2010;96(20):1617-21. doi:10.1136/hrt.2010.198804
2. Ludvigsson JF, Andersson E, Ekbom A, et al. External review and validation of the Swedish national inpatient register. *BMC Public Health*. Jun 09 2011;11:450. doi:10.1186/1471-2458-11-450
3. Ludvigsson JF, Almqvist C, Bonamy AK, et al. Registers of the Swedish total population and their use in medical research. *Eur J Epidemiol*. Feb 2016;31(2):125-36. doi:10.1007/s10654-016-0117-y
4. Costa F, van Klaveren D, James S, et al. Derivation and validation of the predicting bleeding complications in patients undergoing stent implantation and subsequent dual antiplatelet therapy (PRECISE-DAPT) score: a pooled analysis of individual-patient datasets from clinical trials. *Lancet*. Mar 11 2017;389(10073):1025-1034. doi:10.1016/S0140-6736(17)30397-5
5. Urban P, Mehran R, Colleran R, et al. Defining high bleeding risk in patients undergoing percutaneous coronary intervention: a consensus document from the Academic Research Consortium for High Bleeding Risk. *Eur Heart J*. Aug 14 2019;40(31):2632-2653. doi:10.1093/eurheartj/ehz372
6. Yeh RW, Secemsky EA, Kereiakes DJ, et al. Development and Validation of a Prediction Rule for Benefit and Harm of Dual Antiplatelet Therapy Beyond 1 Year After Percutaneous Coronary Intervention. *JAMA*. Apr 26 2016;315(16):1735-49. doi:10.1001/jama.2016.3775
7. Matteau A, Yeh RW, Camenzind E, et al. Balancing Long-Term Risks of Ischemic and Bleeding Complications After Percutaneous Coronary Intervention With Drug-Eluting Stents. *Am J Cardiol*. Sep 1 2015;116(5):686-93. doi:10.1016/j.amjcard.2015.05.036
8. Joyner CD, Peters RJ, Afzal R, et al. Fondaparinux compared to enoxaparin in patients with acute coronary syndromes without ST-segment elevation: outcomes and treatment effect across different levels of risk. *Am Heart J*. Mar 2009;157(3):502-8. doi:10.1016/j.ahj.2008.10.028

© 2022 Simonsson M et al. *JAMA Network Open.*
